# Root Treatment: A Positive Method

**Published:** 1899-03

**Authors:** F. Milton Smith

**Affiliations:** New York City


### ROOT TREATMENT: A POSITIVE METHOD.[1]

BY F. MILTON SMITH, D.D.S., NEW YORK CITY.

SINCE so much has been said upon the treatment of pulpless teeth, it will not be thought strange if the gentlemen present do not enthuse at the mention of the subject.

More time has been given to the discussion of this subject than

---

[1] Read before The New York Institute of Stomatology, November 1, 1898.

to almost any other in which we as a profession are interested. Notwithstanding this, if we judge by the reports of society transactions, and by the articles published in our journals, it would seem as though we had made almost no progress in the treatment of pulpless teeth during the past thirty years.

On page 375, "Harris's Principles and Practice," edition of 1871, we find the following directions for treating pulpless teeth, as practised by Professor Gorgas, of the Baltimore College of Dentistry:

"Remove carefully all diseased and decomposed matter, *mechanically cleansing* the root thoroughly." (Italics are ours in place of his more lengthy description as to how he does it.) "Syringe out all loose particles with tepid water, dry the canal thoroughly, saturate a piece of floss silk with creosote, and pass to the end of the canal; seal up cavity for twenty-four hours, then examine, and, if necessary, repeat the dressing. When not the slightest odor of purulent secretion is perceptible, fill the root temporarily, and wait for a week. Not infrequently it is necessary to repeat this course of treatment several times. In one case two months elapsed before we felt warranted in filling the tooth."

The writer of this paper knows of one case where an eminently respectable dentist of this city treated such a case for more than eighteen months.

With some slight and apparently immaterial changes as to drugs, the writer is inclined to think this is considered orthodox treatment by very many of our best men to-day.

We have personally removed many such temporary—or, as some men term them, permanent—root-fillings of cotton, silk, etc., during the last three years, the last one only a few days since.

In a paper read before this society, our highly esteemed friend, Dr. Louis Jack, of Philadelphia, in describing his treatment of pulpless teeth, says, "When there is no fistula, the difficulty of disinfection is increased. My plan is to expect a correction of the conditions by the slow admission of a disinfectant, which in its nature is not irritating. For this purpose I loosely fill the canal and pulp-chamber with a solution of aristol, and fill the outer cavity around a root-filling instrument of such size as will permit the effusions forming at the apical region to escape through the small opening left on the withdrawal of the broach. I then *repeatedly* dress it in this manner [the italics are ours], making the vent or opening smaller each time. Then [as we suppose when he deems it safe] I temporarily fill the canal tightly with a thread dipped in aristol as a tentative measure."

If our neighbors have removed as many root-fillings of our making, in order to relieve suffering, as we have silk, cotton, and other absorbents for the same purpose, we are willing to admit without further controversy that our method is a failure. If our patients do have serious trouble, they succeed much better in keeping it from us than they used to in the good old days, when we filled temporarily with cotton, string, silk, etc., for then we could not eat our meals in peace, nor could we always get an uninterrupted night's sleep.

We find in our reading that it is considered very bad practice, in opening a devitalized tooth, to immediately remove all the contents of the pulp-canal, lest in our attempt to do so, we force septic material through the end of the root and so produce trouble. We suppose this is why Dr. Jack recommends a slow admission of disinfectant, by loosely filling the canal with aristol.

Dr. J. Foster Flagg, in the last article we read from his pen, says, "I regard it as a matter of first importance that the apical foramen shall not be passed; above all, enlarged." With the latter suggestion we heartily agree. As to the extreme danger of passing the foramen we are not so confident.

In this society, at the October meeting, as we remember it, Dr. J. Morgan Howe cited a case where he had opened a central incisor in which the pulp had been for a long time dead. If we are not mistaken, he said he was very careful not to go up more than two-thirds of the way to the end. Notwithstanding this, an abscess developed inside of forty-eight hours.

It has been a matter of great interest to us that, in many cases cited by our ablest and best men, where they caution us to be extremely careful not to go to the end of the root, they report serious trouble following their most careful efforts in that direction.

In a paper by Dr. Howe, also read before this society, he most appropriately sets forth our relation to the proper treatment of roots, in these words, "The progress which we cannot withstand is demanding of us to-day that we shall make pulpless root-canals inoffensive, not occasionally, and with much labor, endurance, and expense, but that we shall do it often, quickly, and at a minimum of expense."

This, it seems to us, is a fair statement. Your essayist is very glad that, as far as he knows, none of our leading men have gone so far as to say that it is not possible to go to the extreme end of these roots, thoroughly disinfect, render aseptic, fill the canal at the same sitting, and have the operation successful. Had any one of many we might name seen fit to do so, we should approach the matter with even more caution than we now do. Since they have not absolutely denied it, we desire to raise the question, for we believe it can be done in almost every case.

Far be it from the mind of your essayist to come before this company of earnest, faithful men, some of whom were in practice before he saw the light of day, and whose shoe-latchet he is not worthy to unloose, in any boastful spirit to tell of the great things he can accomplish. Nor is he unmindful of that word of caution given to one of old,—"Let not him that girdeth on his harness boast himself, as he that putteth it off." Nevertheless, he cannot be rid of the conviction that he is not a true man who, having received a suggestion from another which has lifted a great burden from his shoulders, does not seek to lead out into the light some other. Nor is he a brave man, even though he fears not the cannon on the battle-field, who from fear of adverse criticism refuses to put forth his best effort to transmit to others the benefit he has received.

We have sometimes failed in accomplishing what another has claimed to be an easy matter, because that one has omitted from his description some detail which he took for granted we were conversant with. This is our apology for more minuteness in describing our method than some before us may think necessary.

Our treatment being almost without exception the same, in all cases of pulpless roots, we shall not classify the different conditions found, which are familiar to all.

Unless the tooth is so sore as that to work on it would produce great discomfort, we at once adjust the dam if possible. We consider this very important, as it leaves us the free use of both hands, with no danger from the saliva. We then open up the pulp-chamber so that we can get directly at the roots; not one, but all. This sometimes necessitates the removal of a large portion of the crown, but we had much rather lose three-fourths of the crown than save nine-tenths and have it in such a condition that our patient dare not bite on it; for in the former case we can supply the crown, but we cannot supply the lack caused by not being able to get at pulp-canals directly.

Therefore, with the old farmer who advised his son to get rich honestly if he could, but get rich, we say, most emphatically, get direct access. Next, remove everything you possibly can from the roots, going to the extreme end if you can get there. We have been two hours getting there sometimes. It is not our practice to enlarge the canal, except sufficiently to get a good entrance. Unless the root is extremely foul we rarely use an antiseptic until we have the root mechanically cleansed. After removing all *débris* possible, if there is a discharge of pus or moisture, we very patiently absorb it with paper wound around broaches, removing each piece after using. If we can now get the root dry (kindly note the provision), we expect to fill it permanently at that sitting, no matter what the previous condition, provided it is not too sore to work upon. If the root is causing pain, we open it freely to give relief, and defer the operation to a subsequent sitting. We said we expect to fill the root at once if we can get it dry. We make this exception because we have found, during ten years past, probably six or eight lateral incisors where the accumulation of a purulent fluid resembling more the serum of the blood than real pus has been so abundant that we have not been able to get the root dry after patiently working one hour. Usually even these can be filled at the second sitting.

Reference has been made to Dr. Flagg's caution not to go through the foramen. Regarding this we would say that we never feel absolutely safe unless we have gone through with a fine broach, for then we feel quite confident our remedy will reach the proper place. We firmly believe we have not lost three teeth in ten years where we have been able to get our medicine through the foramen and have filled the root clear to the end. Having dried the root and, if possible, passed our fine broach through it, we now insist that a messenger shall go up through the end and throttle those little fellows who are up there determined to make trouble. For upward of three years our sheet-anchor was chloride of zinc, ten-per-cent. solution, followed with this root-filling: gutta-percha dissolved in chloroform to the consistency of cream, in which a liberal supply of iodoform powder is mixed. We think the exact quantity not material, but should use probably one drachm to one ounce of gutta-percha solution. Since iodoform is not perfectly soluble in chloroform, we stir the mixture before using. Although the zinc chloride can be depended on to do the work effectually, we found it unnecessarily severe, and for seven years we have used carbolic acid instead.

Whether we awakened those micro-organisms or not by going through the foramen with our instruments, or whether the fresh air we allowed to pass up when we opened the basement door did, "deponent saith not;" it suffices to say that they are there at this stage. I think all will agree on that point. As Josh Billings used to say in regard to some dogs that kept him awake, "they need something, probably killing."

To get our carbolic acid where we want it, we often place a drop in the root, then place a piece of softened gutta-percha in the cavity, and with a burnisher force it into the root.

As to the old fogyisms of using carbolic acid and iodoform, we simply say that with us they accomplish the purpose. So long as they do we shall use them.

When we are satisfied that our carbolic acid has gone where we want it, we dry the root as thoroughly as we can, after which we pump our gutta-percha solution into it, following with shreds of bibulous paper dipped in the solution and packed as thoroughly in the root as we know how. If the root-canal is so small that we are unable to carry these shreds of paper, we, after pumping the mixture in the root with a fine broach, carry a piece of eighteen-carat gold wire as far up as we can. We have some of this wire, about three-one-thousandths of an inch in diameter, which the late Mr. R. S. Williams drew out for us some years since.

Dr. S. J. Perry, of this city, mentioned a similar plan some years ago, but, as we remember, he takes larger wire and files one end to a very fine point.

If the foramen is enlarged, we run a hooked broach through it after placing on the shank of the broach a piece of rubber dam. Having shoved our broach through, we draw it back till the hook catches on the edge of the root; we then shove our rubber up till it touches the cutting edge of the tooth, then withdraw the broach carefully, when we have the exact measurement of the root. This plan was, I think, first suggested by the late Dr. Atkinson.

Having ascertained the length of the root, we take a canal-plugger, place on the end of it a piece of softened gutta-percha about as large as will close the foramen, having first made a mark on the shank of our plugger the exact distance from the extreme end of the gutta-percha that our root is long. After placing our solution of gutta-percha in the root, we carry our root-plugger to the end, leaving the gutta-percha there or stopping when our mark on the instrument is even with the cutting edge of the tooth. We then fill the balance with paper dipped in the solution, as before mentioned, finishing at the outer third with oxyphosphate.

In cases involving a fistulous opening we have often pumped our gutta-percha solution through the fistula, but have never had trouble therefrom.

We find a few roots where the canals are so fine we cannot get even our gold wire into them. These we do not fill except as we can get a little of our solution into them.

It is our custom, after treating and filling a root, to say to the patient that they will have some quite severe pain for a few hours following the treatment. Quite often this prediction is not fulfilled, in which case they are agreeably disappointed; if it is, they are not frightened, and bear it until it subsides.

At the risk of wearying you, we will describe a few cases in which we have pursued the treatment laid down.

In August of 1888 a lady patient came to us recommended by her dentist out of town, she having removed to this city. Previous to his recommending her to us her dentist had drilled into the pulp-cavity of the superior right second bicuspid, to relieve the intense suffering incident to an incipient abscess.

Having practised according to the orthodox style, he very candidly told her that since she lived at so great a distance from him, and it would be necessary for her to have it treated many times, it would be to her advantage to have it treated nearer home. He then recommended your essayist. At the first sitting we treated and filled the root. Severe pain followed, but gradually subsided after a few hours.

The next day the cheek was considerably swollen, but the pain had almost entirely disappeared, and in two or three days all swelling had gone. This patient subsequently, by mutual understanding with her dentist, became our patient, and we have seen her, almost without exception, every year since, and up to last May the tooth had not given a moment's discomfort.

In 1891 the best woman on this continent (of course, excepting the wives of the other gentlemen present) complained of the superior left central incisor giving quite a little pain upon her passing from a warm room to the cold air; and *vice versa*. Upon examination we diagnosed a devitalized pulp. We opened the tooth with some hesitancy, remembering the oft-repeated experiences of the days gone by, and treated and filled it at once. Since the second or third day after the treatment this tooth has been perfectly comfortable.

The following year your essayist developed an abscess, which formed and discharged through the gum from the second upper right bicuspid. He consulted Dr. A. J. Reinhold, then of this city, who had practised this method for several years, and who first suggested it to us. He opened into the root, finding it filled with a fluid almost black in color, treated and filled as before described, and at the same sitting placed in it an excellent gold filling, which may be seen by any gentleman present who desires at the close of the meeting. The fistula did not heal for nearly two weeks, when, upon a portion of the root-filling discharging from the gum through the fistula, it healed perfectly and has never recurred. The time consumed in opening up, treating, and filling the tooth with gold was three hours.

In 1893 a lady came to us suffering with a lower sixth-year molar which she said she had hardly dared to bite on since it was filled, a year previously. We removed an oxyphosphate filling, found some loose cotton in the pulp-chamber, and some iodoform powder in the chamber and opening to roots. We opened it up, but did not get it sufficiently over the soreness for several days so that we could work at it without great annoyance to the patient. In a few days, however, we thoroughly cleansed and treated and filled it, after which the patient could use it with perfect comfort.

We found, also, in this mouth an upper bicuspid which the patient assured us had been open for two years, three dentists having given it up because a filling could not be tolerated. We found it in an unusually foul condition, and immediately treated and filled it, since which, up to a short time ago, when we last saw the patient, it has been perfectly comfortable. These two teeth we looked upon as being as unpromising as any we have ever been called upon to treat.

In 1894 a gentleman came to us who had been treated by a dentist whose reputation as a successful operator in this city is second to none. He wanted us to examine the upper left first molar, which had a fistulous opening, discharging in the roof of the mouth. The patient stated that it had been repeatedly treated some six months previously.

We removed an oxyphosphate filling, and found a very large quantity of cotton in the palatal root in an extremely foul condition. We said to this patient, "You have been treated by a man whose reputation is absolutely first-class. He has treated you after the orthodox plan, and has failed. I can do no worse than he has done. Let us try another plan, and if we fail, have the tooth extracted." He consented. We found the foramen of the palatal root much enlarged. We treated but once, and filled roots and crown. The fistula immediately healed, leaving quite a cicatrix, and had not returned up to last winter, when we saw the patient.

The same year a lady came to us with a central incisor badly abscessed, and with considerable of the process, including the septum between it and the lateral, gone.

We found an opening in the palatal surface, and removed from the root one of the rankest pieces of cotton or silk string it has ever been our fortune to meet. We immediately cleansed, treated, and filled this root and tooth, the abscess at once healed, and the tooth, which had been quite loose, became comparatively firm. The dentist who had treated this tooth assured the patient that, owing to her general systemic condition, she could never have a dead tooth filled successfully.

One of our most recent cases, and we are done with this part of our story. A gentleman came to us with a lower bicuspid badly broken away. We found the root filled. As he said he had had no trouble with it, we concluded to trust it. Before twenty-four hours had elapsed we regretted it. The crown, a gold one, had been set but a few hours when it began to give pain. In about forty-eight hours we removed the crown and found the root filled with cotton. After removing the cotton and running a broach down, we got two or three drops of pus, which gave instant relief. The following day our patient left town for a week, so it remained open. On his return we found no pus, but a very bad odor. We cleansed, treated, and filled the root at once. Considerable swelling followed, but no severe pain. In four days the swelling had subsided, and we believe our patient will have a comfortable tooth for many days.

The foregoing we have taken at random from our experience, and believe they fairly represent our every-day work.

In summing up, we would say that we regard as essential to success in root treatment, first, the rubber dam; second, free direct access; third, thorough cleansing mechanically and antiseptically; fourth, getting the antiseptic through the root; fifth, perfectly filling the root immediately upon getting an aseptic condition with an antiseptic root-filling; sixth, sufficient confidence in the method used to insure thorough work, and the minutest attention to details.

Given these conditions, we believe that any careful operator who treats and fills roots at once will succeed far beyond the one who keeps his roots under prolonged treatment. We do not claim to offer a scientific reason for our success, but we have a theory that to very carefully open a root and remove two-thirds of the canal contents, after the bacteria have been awakened in the other third and beyond the root, is to invite the trouble we seek to avoid. On the other hand, we believe that where we have just opened the tooth, and set forces at work which are bent on its destruction, we ought at once, if possible, to get our antiseptic not only in the root, but also as much farther as the mischievous effects have gone. After we have done this, and before more micro-organisms have come to life, we ought to forestall them by filling the root so thoroughly with an antiseptic material that there will be no home in which they may abide.

Some may ask the question, "Why does the essayist come before this society in the *rôle* of an empiric. We are scientific men, and unless scientific reasons can be given us for methods advocated we cannot be expected to accept them." Our answer is, Whether we will or no, with all our science, we are still and always will be empirics.

What we believed was scientific yesterday, and in our pride followed because it was scientific and successful, to-day we find was empiricism, because we have gone just a step farther. We continue to follow that method of practice to-day because it brings good results, and, forgetting our boasting of yesterday, we still call ourselves scientists. To-morrow will bring us a step farther, when we shall look upon the practice of *to-day* as empiricism; and so each day we may, if faithful, make just a little progress, until that day when "the grinders cease because they are few, and those that look out of the windows be darkened," and the silver cord is ready to unloose and the golden bowl to break. Then we shall look back upon all our attempts to answer the *ultimate* why? and say, "The wind blew where it listed, and we heard the sound thereof, but could not tell whence it came nor whither it went."

Until that time let us seek all the scientific light we may, forgetting not that some methods, the working of which we do not fully understand, may be very helpful to humanity.

